# STIM1-Orai1 interaction mediated calcium influx activation contributes to cardiac contractility of insulin-resistant rats

**DOI:** 10.1186/s12872-022-02586-w

**Published:** 2022-04-05

**Authors:** Aysegul Durak, Yusuf Olgar, Kardelen Genc, Erkan Tuncay, Fırat Akat, Sinan Degirmenci, Belma Turan

**Affiliations:** 1 Department of Biophysics, Faculty of Medicine, Ankara University, Ankara, Turkey; 2 Stem Cell Institute, Ankara University, Ankara, Turkey; 3 Department of Physiology, Faculty of Medicine, Ankara University, Ankara, Turkey; 4 Department of Biophysics, Faculty of Medicine, Eskisehir Osmangazi University, Eskisehir, Turkey; 5 Department of Biophysics, Faculty of Medicine, Lokman Hekim University, Ankara, Turkey

**Keywords:** SOCE, Cardiac, Calcium, Metabolic syndrome

## Abstract

**Purpose:**

Metabolic syndrome (MetS) became a tremendous public health burden in the last decades. Store-operated calcium entry (SOCE) is a unique mechanism that causes a calcium influx, which is triggered by calcium store depletion. MetS-induced alterations in cardiac calcium signaling, especially in SOCE are still unclear. Therefore, we aim to examine the possible role of SOCE and its components (STIM1 and Orai1) in the MetS-induced cardiac remodeling.

**Methods:**

We used male, adult (12 weeks) Wistar albino rats (n = 20). Animals were randomly divided into two groups which were: control (C) and MetS. We gave 33% sucrose solution to animals instead of water for 24 weeks to establish MetS model. In the end, papillary muscle function was evaluated, and various electrophysiological analyses were made in isolated cardiomyocytes. Additionally, STIM1 and Orai1 protein and mRNA expressions were analyzed.

**Results:**

We observed a deterioration in contractility in MetS animals and demonstrated the contribution of SOCE by applying a SOCE inhibitor (BTP2). Calcium spark frequency was increased while its amplitude was decreasing in MetS hearts, which was reversed after SOCE inhibition. The amplitude of transient calcium changes in the MetS group was decreased, and it decreased further BTP2 application. Both protein and mRNA levels of STIM1 and Orai1 were increased significantly in MetS hearts.

**Conclusion:**

Current data indicate the significant contribution of SOCE to cardiac calcium handling in the MetS model. We think MetS-induced SOCE activation is a compensation mechanism that is required for the continuum of proper cardiac functioning, although the activation can also cause cardiac hypertrophy.

**Supplementary Information:**

The online version contains supplementary material available at 10.1186/s12872-022-02586-w.

## Introduction

Metabolic syndrome (MetS) is a cluster of metabolic disorders such as glucose intolerance, insulin resistance, abdominal obesity, dyslipidemia, and hypertension [1]. In 1988, Reaven proposed a model for this constellation and named it ‘Syndrome X’ [2]. Until today, respected clinical institutions made significant alterations both in definition and in diagnostic criteria of MetS. Currently, physicians declare metabolic syndrome if three or more of the following five criteria are met: wide waist circumference, high triglycerides, low HDL-cholesterol, high blood pressure, high fasting blood sugar, and insulin resistance [3]. Each component of MetS is a separate risk factor for cardiovascular disease (myocardial infarction, and stroke, etc.), and they act synergistically [4–6].

Store-operated calcium entry (SOCE) is the process by which the depletion of endoplasmic reticulum calcium stores causes an influx of calcium ions across the plasma membrane [7]. SOCE is the primary calcium entry pathway for the non-excitable cells, but it is also present in the excitable tissues like skeletal or cardiac muscles [8, 9]. The channels which contribute to SOCE are defined as the calcium release-activated calcium (CRAC) channels, and they are first detected in T lymphocytes and mast cells [10–12]. Interaction between stromal interaction molecule (STIM1), embedded in the ER-membrane and Orai1, residing in the plasma membrane forms a calcium selective channel complex which is the primary contributor of SOCE. At first, it was thought that the calcium ions which enter the cell by SOCE were taken directly into the sarcoplasmic reticulum (SR) and stored, but now we know that these ions enter the cytosol and induce the calcium-dependent molecular pathways or affect the contractile dynamics [13].

SOCE was first demonstrated and more evident in neonatal cardiomyocytes because CRACs are abundant in this cell type [14–17]. Since the number of CRACs in cardiomyocytes decreases with age, the contribution of SOCE to total cardiac calcium homeostasis is also decreased. Yet still, SOCE is an important mediator of calcium homeostasis because Beridge et al. argued that even minor changes in calcium balance can gradually alter cardiomyocyte function in time [18]. Together with functional alterations, calcium signals may initiate the formation of hypertrophy in cardiomyocytes [19].

Although there is a fair amount of information about the effects of SOCE on cardiac calcium homeostasis, many questions are still rising about the role of STIM1 and Orai1 proteins in cardiac pathologies. Some studies report that STIM1 expression was increased in hypertrophic hearts [20–23], and suppression of STIM1 prevented the formation of hypertrophy in the heart [21, 24]. Furthermore, clinical studies emphasize that alterations in SOCE cause cardiac functional abnormalities, and highlight the importance of SOCE for human health [25, 26].

With membrane deporization in heart cells, the L-type calcium channel (LTCC) is activated, resulting in calcium entry into the cell. Calcium entering the cell activates the ryanodine receptor (RyR2), which is an intracellular calcium channel, and initiates the release of large amounts of calcium from the stores in the sarcoplasmic reticulum, promoting cell contraction. This mechanism is called calcium-induced calcium release (CICR) [25]. CICR is mainly induced by the voltage-gated calcium channels (VGCC). Therefore, it is the main determinant of cardiac contractile activity. Further research is needed to designate the contribution level of SOCE and its role in the MetS-induced cardiac pathologies. Therefore, in the present study, we aim to examine whether SOCE contributes to MetS-induced cardiac dysfunction. In this context, we are planning to measure the expression levels of STIM1 and Orai1 and observe their contribution to the contractile activity of papillary muscle strips. We also tested a comprehensive electrophysiological evaluation to understand the calcium channel dynamics in the control and MetS groups.

## Methods

### Ethical approval

All procedures were handled under the Guide for the Care and Use of Laboratory Animals published by the US National Institutes of Health (NIH publication No. 85-23, revised 2011) and we also followed the ARRIVE Guidelines [27]. The presented experimental procedures were examined and approved by the Committee on the Local Ethics of Animal Experiments of Ankara University (Approval number: 2019-11-105).

### Animals and experimental groups

All animal experiments were conducted in the animal laboratory unit located in the Ankara University, Faculty of Medicine, Biophysics Department.

Male, adult (12 weeks old, 200–220 g) Wistar albino *(Rattus norvegicus)* rats were obtained from Ankara University Experimental Animals Breeding and Research Laboratory (n = 20). Sample size decided according to previous mortality rates in our lab. Animals were kept in constant temperature (22–24 °C), humidity (50–55%), and 12 h light/12 h dark cycle. Animals had ad libitum access to standard rat chow and tap water.

After one week of adaptation, animals were randomly divided into two groups:

1. Control group (Con) (n = 10)
2. Metabolic Syndrome Group (MetS) (n = 10)

Body weights and blood glucose levels of animals were measured. All animals were euglycemic prior to experiment (data not shown). Because of technical reasons, it is impossible to make functional (papillary muscle function) and electrophysiological analyzes on the same heart sample. Therefore, we used separate animals for functional (n = 6) and electrophysiological (n = 4) analyses. There was not any mortality during animal experiments.

### Induction and validation of metabolic syndrome

We followed the methodology of our previous study in which we have successfully established the MetS model in 24-weeks [28, 29]. Briefly, the control group was given ad libitum standard chow and tap water, while the MetS group was given ad libitum standard chow and sucrose (Sucrose, Cat. No.: 107651 Merck Millipore, Germany) solution (33%) for 24 weeks. Bodyweight, fasting blood glucose, serum insulin level, and blood pressure of the animals was monitored to validate the establishment of the MetS model. Moreover, we made an oral glucose tolerance test (OGTT) to evaluate insulin resistance.

OGTT and blood pressure measurements were made on separate days, in the 23rd week (one week before sacrification). One day before OGTT, animals were fasted overnight (12–16 h). We made a tiny incision to the tail for measuring blood glucose by using a glucometer (On Call Plus Glucometer; Acon Labs Inc. USA). The first measurement (0"), is the fasting blood glucose parameter at the same time. We repeated the measurement at 15th, 30th, 60th, and 120th minutes.

The blood pressure of the animals was measured under mild anesthesia (45 mg/kg Ketamine 5 mg/kg Xylazine i.p). Briefly, we attached and inflated a pressure-sensitive tail-cuff to the mid-portion of the tail. The tail-cuff was connected to a digital-analog converter (MP35 Biopac Systems USA). We made five consecutive measurements and took the average. Systolic and diastolic pressures levels were measured and pulse pressure was calculated according to the formula: Pulse Pressure = Systolic Pressure − Diastolic Pressure.

Finally, serum insulin concentrations were measured with a specific ELISA Kit (Insulin EIA Kit; Cat. No.: 589501; Cayman Chemicals; USA). We followed the manufacturer's instructions throughout the assay.

### Papillary muscle function

Rats were anesthetized by sodium thiopental (50 mg/kg; i.p.) The hearts were quickly removed and taken into Krebs–Henseleit buffer (content in mM: 119 NaCl, 4.8 KCl, 1.8 CaCl2, 1.2 MgSO4, 1.2 KH2PO4, 20 NaHCO3, and 10 glucose, pH 7.4) that was pre-aerated with carbogen (95% O2 and 5% CO2). Hearts were weighed, and the heart weight (HW) values were divided to the terminal body weight (BW) to calculate HW/BW ratio. Left ventricular papillary muscle was dissected and connected to the stimulating electrode from one end, and the other end of the muscle was connected to the force–displacement transducer (FT-03, Grass Instruments, Austin, TX). The Krebs–Henseleit solution was kept at a constant temperature (37 °C), and continuously aerated with carbogen throughout the experiments (Tuncay 2020). Papillary muscles were stimulated (frequency: 0.2 Hz) with a special stimulator (Grass S48), for 3 ms to obtain twitches. Twitches were recorded with an amplificatory (Grass P16) and transformed to digital by an analog–digital converter (Adventech PCL-818 PG). The sampling rate was 10 kHz. We analyzed the amplitude of the twitch (mg), and the contraction-relaxation rates (mg/s). These two parameters were normalized to the length of the papillary muscle (mm). After baseline responses were recorded 5 µM BTP2 (CRAC Channel Inhibitor, BTP2, CAS 223499-30-7, Santa Cruz Biotechnology, USA) was applied and all aforementioned analyses were repeated.

### Electrophysiological analysis

#### Isolation of cardiomyocytes

At the end of the 20th week, cardiomyocytes were isolated from the left ventricle of the rats by an enzymatic method. Briefly, hearts were quickly removed under mild anesthesia (30 mg/kg sodium thiopental, i.p.), and placed in an ice-cold, calcium-free Tyrode solution. (The content of Tyrode solution (in mM): 117 NaCl, 5.7 KCl, 3.6 MgCl_2_, 1.5 KH_2_PO_4_, 4.4 NaHCO_3_, 20 HEPES, 10 glucose, and 20 Taurine; pH 7.4). Hearts were attached to a Langendorff apparatus via a cannula inserted from the aorta into the left ventricle. After fixation of the aorta with 3.0 silk suture, hearts were perfused with calcium-free Tyrode solution aerated with 100% O2, containing 1.2 mg/mL collagenase (type II; Worthington Biochemical Corporation, USA) for 25 min at constant temperature (37 °C). After perfusion, the hearts were detached from the apparatus and mechanically dissected in a petri dish. Tissue debris was filtered and isolated single cells were obtained. Cells were adapted to calcium by gradually increasing the calcium concentration (0.1, 0.3, 0.5 mM respectively).

#### Measurement of basal level of free calcium, the transient calcium changes, and sr calcium content

To measure intracellular basal level of free Calcium ([Ca^2+^]_i_), isolated cardiomyocytes were taken into Tyrode solution and incubated with Fura-2 (AM; 4-µM) (Fura-2, AM, cell-permeant, Cat.No: F1221, Invitrogen) for 30 min at room temperature. After 30 min cells washed Tyrode solution (2 × 10 min) to remove the external dye. Intracellular basal [Ca^2+^]_i_ and the transient changes of [Ca^2+^]_i_ changes (under electrical stimulation) were measured by using microspectrofluorometry and FELIX software (Photon Technology International, Inc., NJ USA). The cells were excited with λ = 340–380 nm and emission was taken at 520 nm, the basal [Ca^2+^]_i_ was recorded for 30 s, and then transient [Ca^2+^]_i_ changes was recorded for 200 s by stimulating them with 25–30 V pulses at a frequency of 0.2 Hz (Grass Medical Instruments SMD9F, USA). Cells were exposed to BTP2 (CRAC Channel Inhibitor, BTP2, CAS 223499-30-7, Santa Cruz Biotechnology, USA) (5 µM) for 15 min to inhibit SOCE. Stimulation was ceased during incubation. After inhibition, all aforementioned analyses were repeated. At the end of the experiment, caffeine (10 mM) (Caffeine, C0750, Sigma Aldrich, USA) was administered to measure the SR calcium content.

#### Measurement of calcium sparks

For spark measurements, cardiomyocytes were incubated with Fluo-3 AM (non-ratiometric) calcium dye (Fluo-3, AM, Calcium Indicator, Invitrogen Cat No.: F1241) in Tyrode's solution for 1 h at room temperature. Fluorophore was excited at 488 nm and emission values at 525 nm were recorded in linear scanning mode (x − t mode) using a Leica TCS SP5 laser scanning microscope. The scanning line (512 px) was vertically positioned to the long axis of the cardiomyocytes.Every image was recorded with 63X water immertion (NA = 1.3) objective, in 800 Hz speed at room temperature (20–25 °C). Analysis was performed using Sparkmaster (an ImageJ plug-in) and ImageJ programs. The spark parameters such as, peak amplitude (Δ*F*/F_0_) the time to a maximum value of the signal [time to peak (TP)], full width half maximum (FWHM), and the full duration at half-maximum (FDHM) were analyzed with the analysis program. Spark frequency values were calculated as 100 μm^−1^ s^−1^.

#### Measurement of store operated calcium entry (SOCE)

Store Operated Calcium Entry (SOCE) was recorded as described previously [30]. Briefly, cardiomyocytes were incubated with Fluo-3 AM in Tyrode's solution for 45 min at room temperature. Fluorescence measurements were recorded using a microspectrofluometer and FELIX software (Photon Technology International, Inc., NJ USA) with an emission of 525 nm by stimulating at wavelengths of 488 nm. For SOCE measurement, calcium-free solution containing SERCA blocker thapsigargin (2 µM) (Thapsigargin Cat.No:9033, SigmaAldrich, USA) to block Calcium ATPase, calcium channel blocker verapamil ((±)-Verapamil hydrochloride, Cat. No.: V4629, Sigma Aldrich, USA) (10 µM), and Na^+^/Ca^2+^-exchanger blocker KB-R (KB-R; Cat.No: 7943 Abcam, UK) (10 µM) was used to empty the SR/ER calcium store. After long-term exposure (10 min), we applied Caffeine (10 mM, 3-min intervals) three times to empty SR/ER calcium store (Fig. 3C). Afterward, we measured the fluorescence increase by switching to 1.8 mM Ca^2+^ solution and labeled it as SOCE. The calcium flow from the beginning to the top was taken as SOCE.

#### Measurement of SOCE current using the patch-clamp technique

Electrophysiological parameters of cardiomyocytes were recorded by using the Axoclamp patch-clamp amplifier (Axopatch 200B amplifier, Axon Instruments, USA) at room temperature (23 ± 2 °C). Currents were sampled and digitized at 5 kHz using an analog-to-digital converter and software (Digidata 1200A and pCLAMP 10.0; Axon Instruments, USA). SOCE currents (I_SOCE_) were recorded as described elsewhere [31]. Sarcolemmal I_SOCE_ were recorded using internal solution containing in mM: 130 CsCl, 10 TEACl, 1 MgCl_2_,, 5 MgATP, 10 HEPES, 10 EGTA while free calcium was maintained at 100 nmol/L with CaCl_2_ (pH 7.2). The extracellular bathing solution for I_SOCE_ recordings contained in mM: 140 NaCl, 5 KCI, 1.8 CaCl_2_, 0.5 MgCl_2_, 0.33 NaHPO4, 10 HEPES, 5.5 Glucose, pH was adjusted at 7.4. Sarcolemmmal I_SOCE_ were evoked by applying a descending ramp protocol ranging from + 50 mV to − 110 mV with a speed of 6.25 mV/ms while holding potential was − 70 mV. Calcium depletion from intracellular stores was achieved by applying nominally free calcium bath solution containing 2 µM Thapsigargin (SERCA blocker), 10 µM verapamil (Calcium channel blocker), 10 mM TEA (K^+^ channel blocker), and 10 µM KB-R 7943 (Na^+^/Ca^2+^-exchanger blocker) for 10 min. Then, the specific SOCE inhibitor BTP2 (20 µM) was added to allow the reflow to stabilize. The current after the BTP2 application was subtracted from the current before the BTP2 application to calculate I_SOCE_ (Fig. 2A, B). All currents were divided into cell capacitance and presented as pA/pF.

### Western blotting

Isolated left ventricular cells were prepared as previously described [29]. Briefly, cells were first kept on ice for 10 min with homogenization buffer (250 mM NaCl, 1% NP 40 and 50 mM Tris–HCl; pH 8.0 and 1X PIC). The supernatant was obtained by centrifugation at 12,000 × g for 10 min. The protein concentration of the supernatants was determined with the BCA kit (Biorad Cat.No: #5,000,001, USA). An equal amount of protein was loaded using 8–12% Bis–Tris gels (Life Technologies). Proteins on the gel were transferred to PVDF membrane (Biorad Immun-Blot® PVDF Membrane). We used specific transfer buffer provided by manifacturer Biorad #1,610,778, USA). After transfer procedure, membrane was submerged in the readily-prepared Ponceau S solution [Briefly, Ponceau S dye (Sigma-Aldrich P3504) (0.1% w/v) was dissolved in acetic acid solution (3,5% v/v)] for 1 min and then image of the stained membrane was recorded. Afterward, membrane was incubated with a primary antibody (12 h) against STIM1 (Cell signaling, 9379S, 1:1000), Orai1 (GeneTex, GTX16613, 5ug/ml), and β-actin (Santa Cruz, SC-47,778, 1:500). The membranes were washed and incubated for 1 h with secondary antibodies (Advansta secondary antibodies 1:20.000) and were washed again and incubated for 1 min with ECL (Enhanced Chemiluminescence) and imaging was performed (Azure 300 Chemiluminescent Western Blot Imaging System).

### Measurement of mRNA expression

Total RNA isolation from isolated cardiomyocytes was performed using the RNA isolation kit (Macherey–Nagel, 740.955.10) and purified total RNA was reverse transcribed with the ProtoScript First-Strand cDNA Synthesis-kit (New England Biolabs, E6300S) as described previously [32]. The size and specificity of the primers were checked with the NCBI and ENSEMBL databases. Cyclophilin was used as house-keeping control. The primary sequence for Cyclophilin, STIM1 and Orai1 in heart tissue are given in Table 1. STIM1 and Orai1 normalizations were performed relative to Cycolphilin expression. GoTaq® qPCR Master Mix (Promega, A6001) was used to quantify stranded cDNAs. qRT-PCR was performed using LightCycler 480 (Roche). The fold changes of genes were analyzed based on the comparative (2 − ΔΔCt) method.

**Table 1:** The nucleotide sequences of the primers for STIM1, Orai1 and cyclophilin

Primer	5′-sense primer-3′	5′-antisense primer-3′	Base pairs (bp)
STIM1	GGCATCTTGCTTTGGAACCG	CAATCCGGCAAAACTCTGCT	133
Orai1	GATCGGCCAGAGTTACTCCG	TGGGTAGTCATGGTCTGTGTC	202
Cyclophilin	GGGAAGGTGAAAGAAGGCAT	GAGAGCAGAGATTACAGGGT	211

### Statistical analysis

All data were evaluated by two researchers. Researchers were not blind to the data. We used GraphPad Prism (GraphPad Prism for Windows v5 2007) software for statistical analysis. At first, we performed the Shapiro–Wilk test to all parameters as a test of normality. According to the result, we decided that parametric test assumptions have been met. Independent Samples t-test was used for pairwise comparisons between Con and MetS group while Paired Samples t-test was preferred for comparisons between MetS and MetS + BTP2 groups. Repeated measures ANOVA and Tukey test as Post-Hoc was preferred for OGTT data. p < 0.05 was accepted as statistically significant.

## Results

### Validation of the metabolic syndrome model

To validate metabolic syndrome, we evaluated body weight, insulin levels, oral glucose tolerance test (OGTT), and blood pressure parameters of the animals. We also calculated the heart weight (HW) / body weight (BW) ratio as an indicator of cardiac hypertrophy (Table 2).

**Table 2:** Metabolic parameters of animals ($\hat{x}$ ± SD)

Metabolic parameters	Control	MetS	p value
Body weight (g)	414.00 ± 45.48	490.10 ± 45.23	< 0.0001
HW/BW ratio	3.84 ± 0.34	3.54 ± 0.36	= 0.16
Blood pressure (mmHg)			
Systolic	125.50 ± 7.50	184.90 ± 16.91	< 0.0001
Diastolic	85.15 ± 10.88	115.80 ± 10.10	< 0.0001
Pulse pressure	40.31 ± 8.34	69.13 ± 10.89	< 0.0001
Fasting blood glucose (mg/dl)	72.85 ± 2.30	96.38 ± 13.08	< 0.01
Insulin level (ng/ml)	3.69 ± 1.63	8.02 ± 3.44	< 0.05
OGTT (mg/dl)			
0”	72.85 ± 2.30	96.38 ± 13.08	< 0.01
15’’	111.08 ± 7.97	173.25 ± 31.96	< 0.01
30’’	108.77 ± 11.03	160.38 ± 34.39	< 0.05
60’’	99.92 ± 7.99	125.88 ± 27.60	= 0.15
120’’	85.77 ± 4.82	105.50 ± 17.75	= 0.08

Terminal body weights of the animals in the MetS group were significantly higher than control animals (p < 0.0001). Fasting blood glucose and insulin levels of MetS animals were significantly higher compared to controls (p = 0.0067 and p = 0.017 respectively). Furthermore, OGTT, which is the main indicator of insulin resistance significantly differed among groups (p < 0.0001). There was a marked difference between groups in timepoints 0, 15, and 30 (p = 0.0067, p = 0.0039, and p = 0.0172, respectively). All blood pressure parameters (systolic, diastolic, and pulse pressure) differ among the groups (p < 0.0001 for all), which indicates that MetS elicited a prominent hypertensive effect. Finally, MetS did not alter the HW/BW ratio, which is the primary indicator of cardiac hypertrophy (p = 0.16).

### Basal [Ca^2+^]_i_, the [Ca^2+^]_i_ transients and SR calcium content

**Fig. 1:**
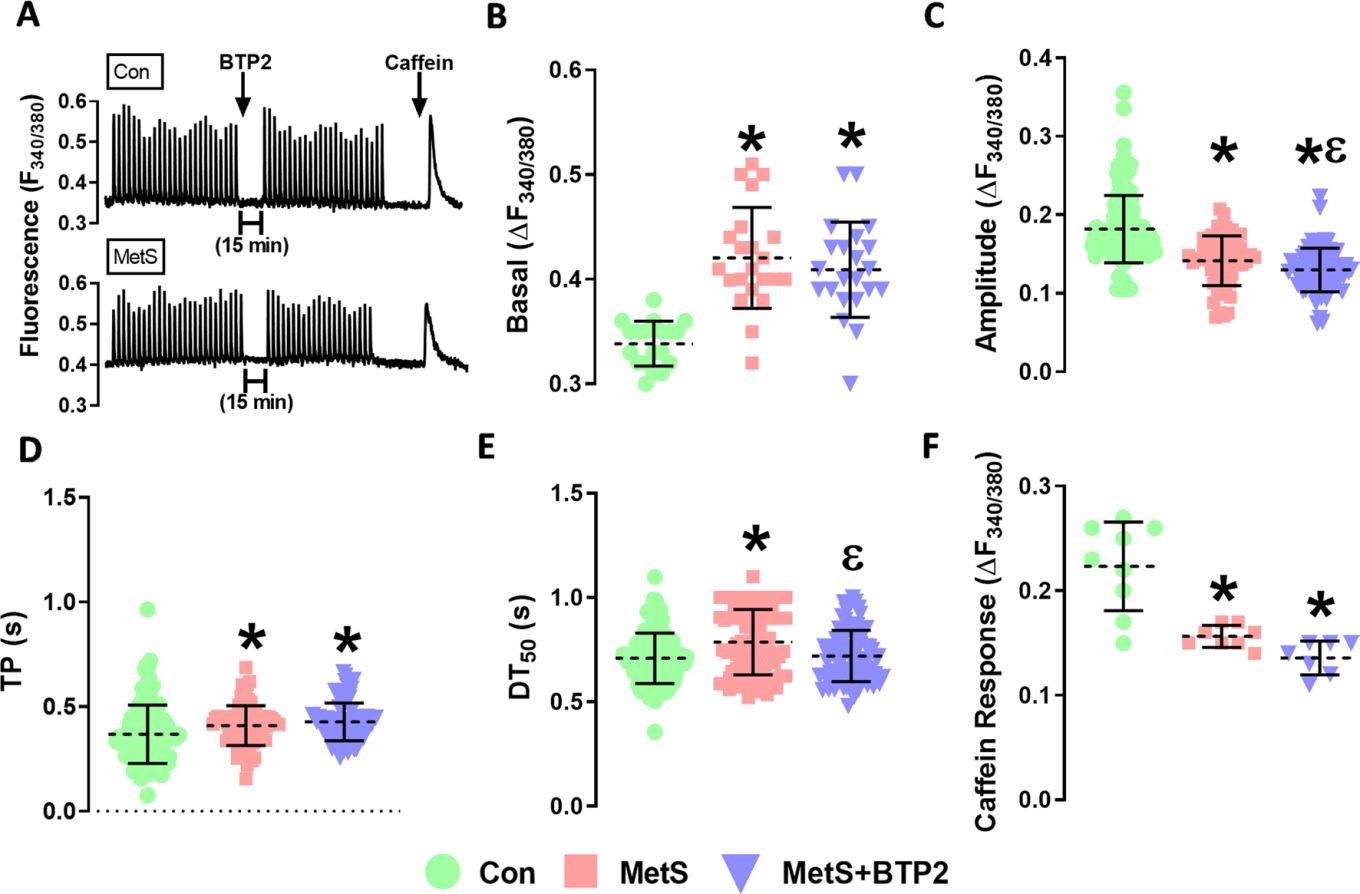
The effect of MetS, and BTP2 on intracellular Ca^2+^ parameters. **A** Representative Ca^2+^ transients. The effect of the BTP2 effect and Caffeine was also demonstrated. **B** Intracellular Ca^2+^ changes in resting-state **C** The peak amplitude of the fluorescences related to the global Ca^2+^ transients are given as F/F_0_ **D** The time to peak fluorescence (TP), **E** The half-decay time (DT_50_), and **F** The caffeine response (n = 22, and 24 cells for CON and MetS, respectively, *:p < 0.05 vs. Con; ε: p < 0.05 vs. MetS)

The effect of MetS and BTP2 on basal [Ca^2+^]_i_, the [Ca^2+^]_i_ transiecalnt parameters (Amplitude, time to peak, and decay time), and SR calcium content (Caffeine response) results were given in Fig. 1. The basal [Ca^2+^]_i_ were found to be significantly higher in the MetS group compared to the control (p = 0.046). Furthermore [Ca^2+^]_i_ did not changed after BTP2 incubation (p = 0.9097) (Fig. 1B).

Transient [Ca^2+^]_i_ changes were induced by electrical stimulation with 25–30 V pulses at a frequency of 0.2 Hz. The mean peak amplitude values in the MetS group significantly decreased compared to the control (p < 0.0001), and after 15 min of BTP2 application (electrical stimulation was ceased during incubation), the amplitude values decreased more after BTP2 application (p = 0.0165) (Fig. 1C). In addition, time to peak (TP), and first half-time for recovery of [Ca^2+^]_i_ transients (decay time; DT_50_) were significantly prolonged (p = 0.019, and p < 0.0001 respectively) and BTP2 administration did not affect TP (p = 0.4852) but decreased DT_50_ (p = 0.0016) (Fig. 1D, E).

Finally, 10 mM caffeine was applied to all cells to evaluate the calcium-content of SR. While the caffeine-induced responses were significantly lower in the MetS group compared to the control (p = 0.07), the BTP2 application did not alter the magnitude of the response (p = 0.1095 (Fig. 1F).

### Evaluation of calcium sparks

**Fig. 2:**
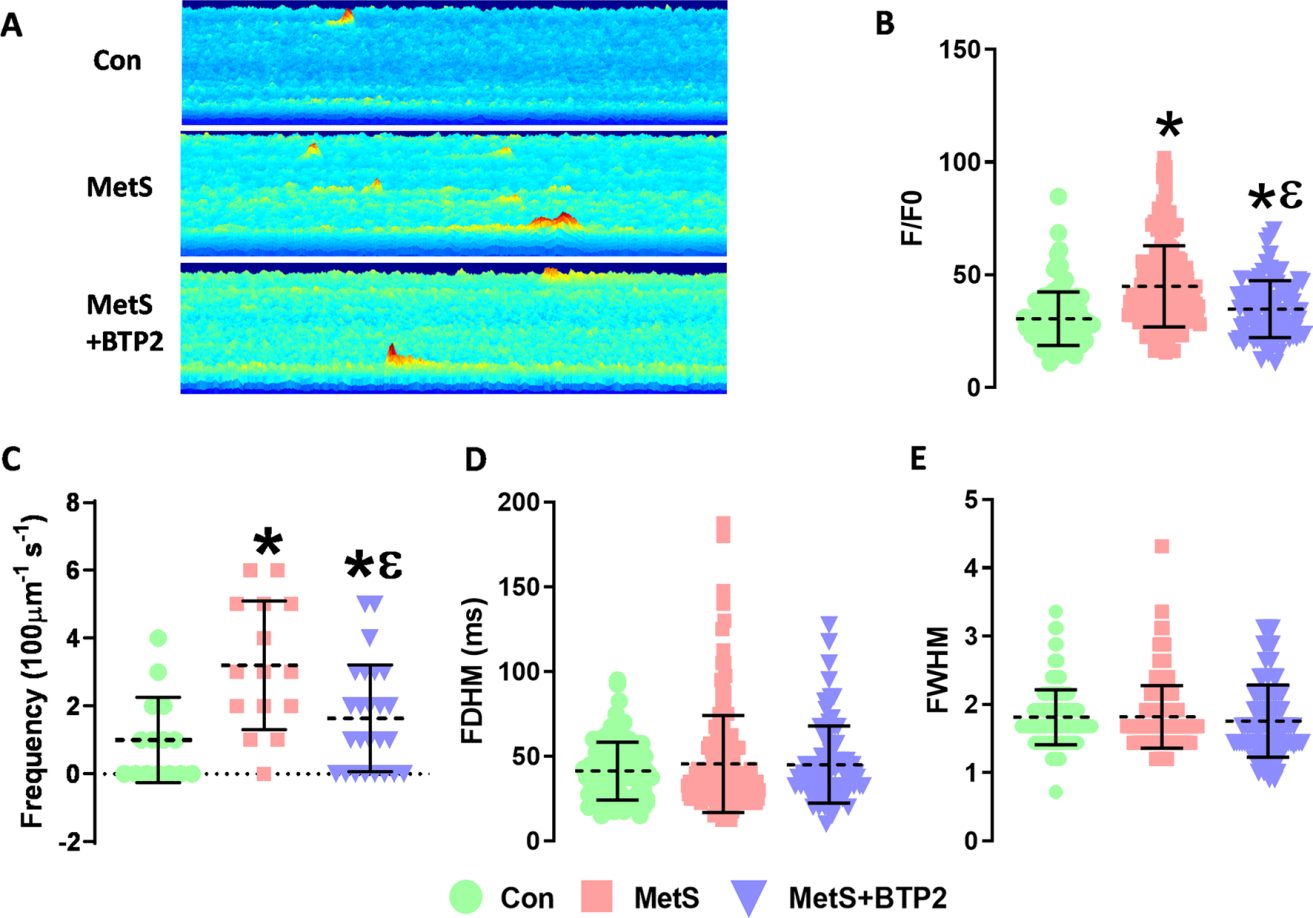
SR Ca^2+^ leakage and effect of BTP2. **A** Representative line scan images from cells loaded with Fluo 3 **B** Peak intensity (F/F_0_) **C** Spark frequency (100 µm^−1^ s^−1^) **D** Full duration at half-maximum (FDHM) **E** Full width half maximum (FWHM) fluorescence changes. *(The recording after BTP2 application was * *taken from the same cell without changing the line scan)* (n = 25, and 30 cells for Con and MetS, respectively, *: p < 0.05 vs. Con, ε: p < 0.05 vs. MetS)

Representative line-scan images of calcium sparks were presented in Fig. 2A. The maximum fluorescence intensity (F/F0) of calcium sparks increased significantly in MetS compared to control (p = 0.0001) and markedly decreased with BTP2 application (p = 0.0001) (Fig. 2B). Moreover, spark frequency increased significantly in the MetS group (p = 0.0008), while the BTP2 application decreased spark frequency (p = 0.0079) (Fig. 2C). The full duration at half-maximum (FDHM) and the full-width half-maximum (FWHM) values did not differ among groups (Fig. 2D, E).

### The effect of metS on SOCE mechanism

**Fig. 3:**
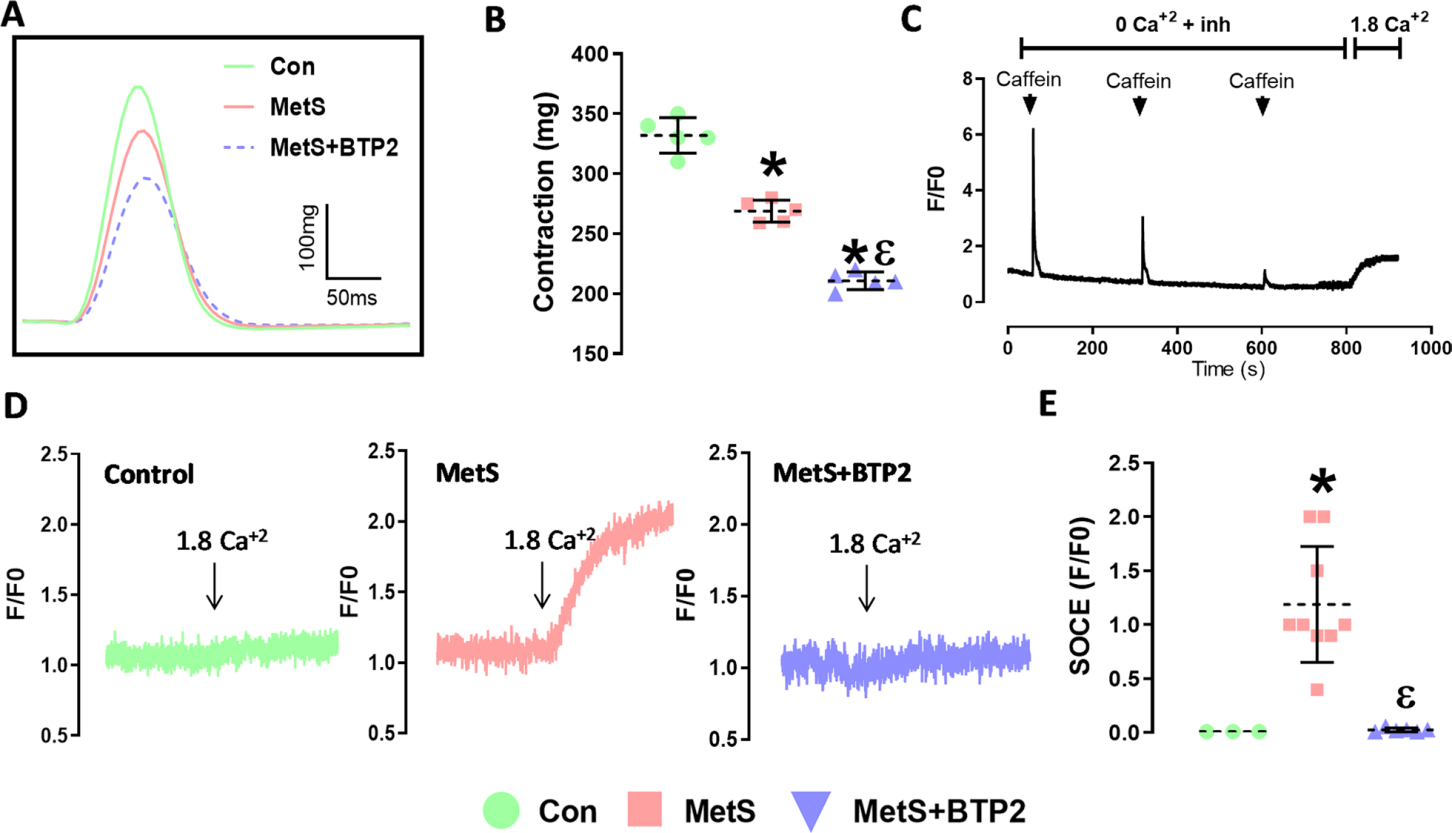
Papillary muscle contraction in rat ventricular strips **A** Representative contraction curves recorded from papillary muscles **B** The net contraction that was recorded in electrically stimulated papillary muscle strips. (n = 4 each **C** Trace shows the preparation steps before SOCE measurement. The cells were first exposed to a Ca^2+^-free bath solution, containing various inhibitors (2 µM Thapsigargin (SERCA blocker), 10 µM verapamil (Ca^2+^ channel blocker), 10 mM TEA (K^+^ channel blocker), and 10 µM KB-R 7943 (Na^+^/Ca^2+^-exchanger blocker), and then caffeine (10 mM, 3 min intervals) was administered to empty SR/ER Ca^2+^ stores. At the end of 10 min, the fluorescence increase was calculated by switching to a bath solution containing Ca^2+^(1,8 mM) **D** Representative SOCE in rat left ventricular myocytes for control and MetS groups. **E** SOCE in rat left ventricular myocytes after SR Ca^2+^ depletion for the control, MetS, and MetS + BTP2 groups. Fluo3 AM dye was used for SOCE measurement. (n = 8,9,6 cells for CON, MetS, MetS + BTP2 respectively) (*: p < 0.05 vs. Con)

Papillary muscle contraction curves were given in Fig. 3A. According to our initial observation, papillary muscle contractility was lower in the MetS group compared to controls, and contractility decreased further after BTP2 application that indicating a significant contribution of SOCE to the papillary muscle contractility of the animals in the MetS group (Fig. 3B). Representative SOCE fluorescence measurement protocol was given in Fig. 3C and also images of SOCE fluorescence and the effect of BTP2 were presented in Fig. 3D. We demonstrated the calcium influx that originated from SOCE was increased significantly in the cells with MetS (p = 0.0042), and it almost completely disappeared after the application of BTP2 (p = 0.0002) (Fig. 3E). SOCE measurements were repeated with a ratiometric calcium dye (Fura-2AM) in another set of experiments, and SOCE peak values were measured significantly high in the MetS group compared to Con (data not shown).

### The effect of metS on SOCE current

**Fig. 4:**
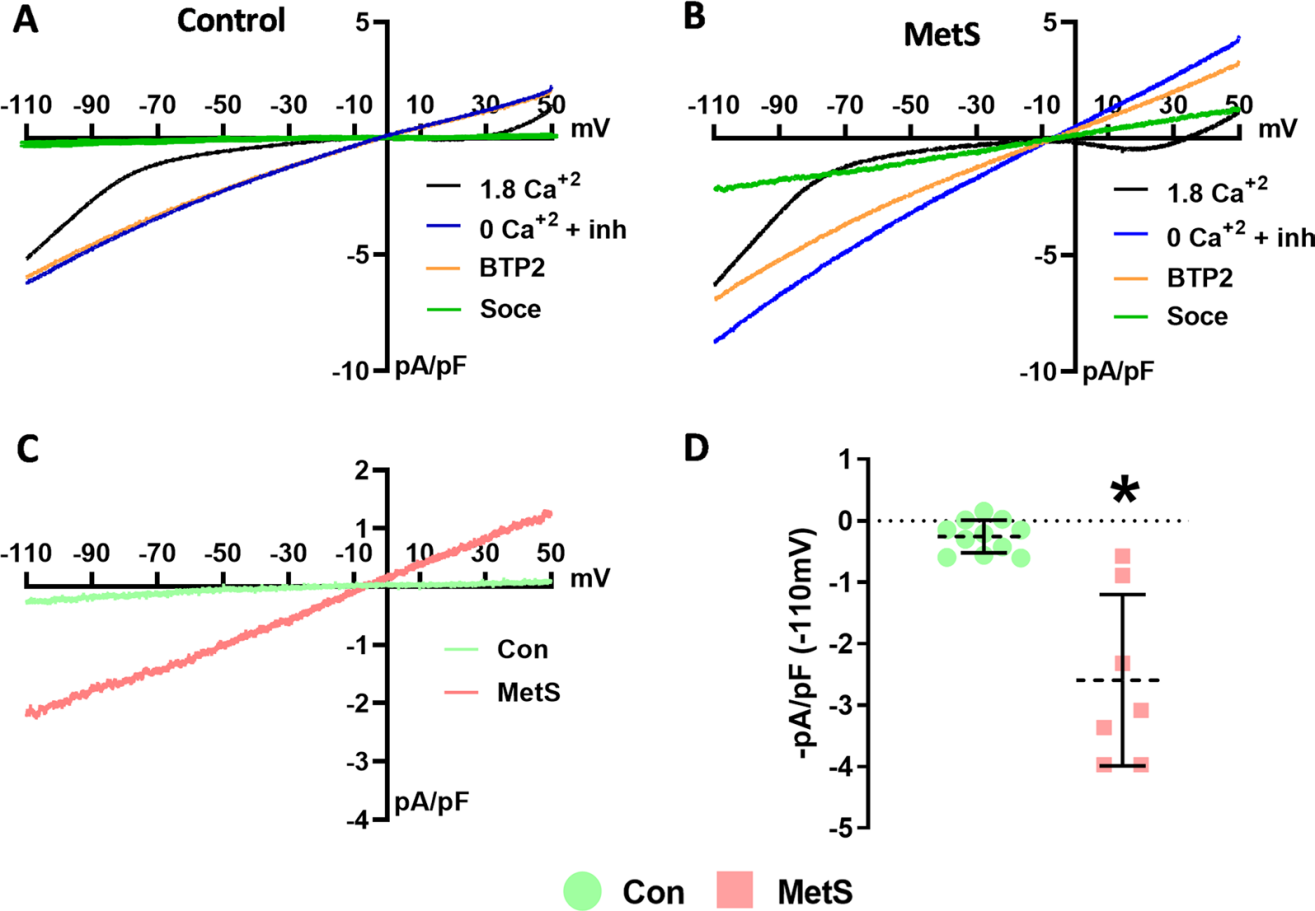
Whole-cell patch-clamp recording of store-operated Ca^2+^ current in rat left ventricular myocytes**. A** Whole-cell patch-clamp current recorded in Con group **B** Whole-cell patch-clamp current recorded in MetS group [Black line: presence of Ca^2+^ (1.8 mM); Blue line: absence of Ca^2+^ (0 mM + 2 µM Thapsigargin + 10 µM verapamil + 10 µM KB-R 7943); Orange line: BTP2 added (selective SOCE blocker, 20 μM); Green line: SOCE current (I_SOCE_) (= Orange—Blue)] **C** Comparison of I_SOCE_ current between Con (black) and MetS (orange) groups. **D** The calculated maximum current of I_SOCE_ (− 110 mV) (n = 11, and 9 cells for Con and MetS, respectively *: p < 0.05 vs. Con)

Whole-cell patch-clamp currents recorded in Con and MetS groups were given in Fig. 4. To obtain SOCE current we subtracted the blue curve from the orange one. In the control animals, the difference (green curve or the SOCE current) was almost non-existent (Fig. 4A). However, SOCE current was prominent in the MetS group (Fig. 4B). Therefore, the results tell us there was a significant increase in SOCE current in MetS animals compared to controls. SOCE currents (I_SOCE_) from both groups of cells were given in Fig. 4C, and the maximum current values were presented as bar-graphs in Fig. 4D. –pA/pF values differed significantly between Con and MetS groups (p = 0,0061).

### Effect of MetS on protein and mRNA expression levels of STIM1 and orai1

**Fig. 5:**
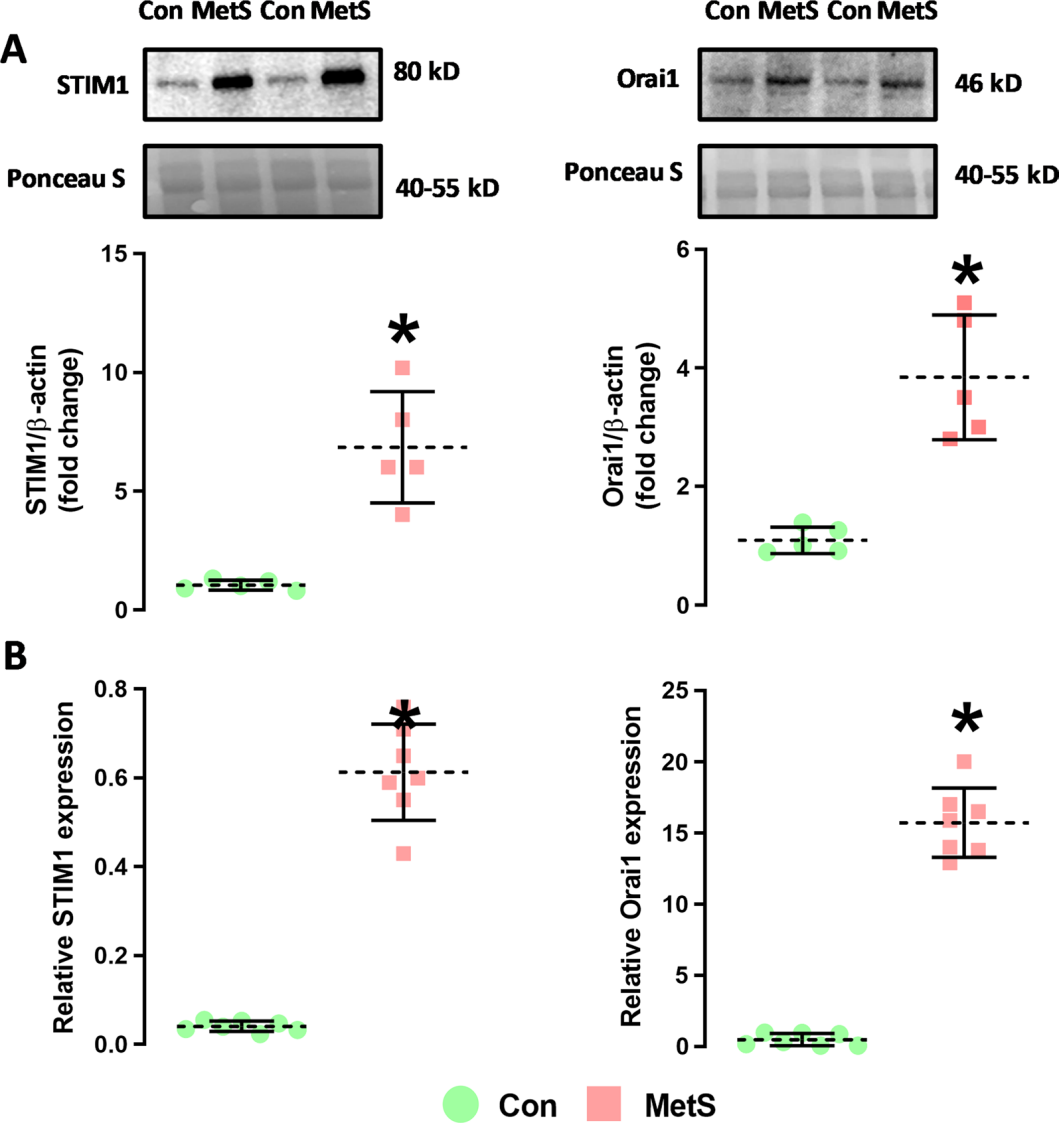
The mRNA and protein levels in isolated ventricular cardiomyocytes. **A** STIM1 and Orai1 protein levels **B** STIM1 and Orai1 mRNA expression levels (*:p < 0.05 vs. Con)

To support the SOCE activity data, we measured the protein (Fig. 5A) and mRNA (Fig. 5B) levels of STIM1 and Orai1 in MetS and Con groups. Protein and mRNA levels of STIM1 and Orai1 from lysates obtained from isolated cardiomyocytes were significantly increased in MetS compared to control animals. Western blot images were shown in Additional file 2: Fig. S2.

## Discussion

Current data explicitly point out the crucial contribution of SOCE to cardiac calcium homeostasis. Both SOCE and the expression of SOCE-related proteins were significantly increased in the MetS group, and this effect also altered the contractile dynamics. Although the deterioration of cardiac contractility and alterations in the calcium-homeostasis was widely demonstrated in various metabolic syndrome models, further studies are needed to establish the crosslink between metabolism and cardiac calcium-homeostasis. According to current results, SOCE seems to become an important candidate for revealing the underlying mechanism of MetS induced cardiac remodeling.

According to the latest guidelines, if three of the following five criteria are met, the patient is diagnosed with metabolic syndrome [3]. We monitored various metabolic parameters of the animals to validate our metabolic syndrome model. Animals in the MetS group had significantly higher body weights and blood pressure levels. Furthermore, OGTT revealed that MetS animals showed prominent insulin resistance, and their serum insulin levels were high compared to control animals. Collectively these results validated that drinking sucrose solution for 24 weeks induced a MetS in Wistar rats.

Alterations in cardiac contractility due to mishandling of the [Ca^2+^]_i_ have been previously reported in the rats with MetS [28, 33, 34]. Moreover, depressed cell contraction and alterations in intracellular calcium-homeostasis have been widely documented in cardiomyocyte levels in the sucrose-fed rat models [26, 28, 34–38]. However, the effect of SOCE on contractility in hearts with MetS was not investigated in studies. In our study, it was shown that the decreased contraction forces in hearts with MetS were further reduced when SOCE was suppressed using BTP2 (selective SOCE inhibitor). Previous studies that used various high-sucrose feeding models, repeatedly demonstrated impairment in SERCA2a activity [26, 28], an increment in RYR2 phosphorylation [25, 28], thus a disruption in intracellular calcium regulation. The derangement in RYR2—SERCA2a balance causes SR-load alterations and impairment in contractile function [25, 26, 28, 29, 34]. Papillary muscle contractility was impaired in the MetS group, which is consistent with the literature. Furthermore, Caffeine response, which indicates the abundance of calcium inside the SR, was significantly reduced in MetS animals.

The increase in diastolic [Ca^2+^]_i_ contributes to the deterioration of the relaxation rate in cardiomyocytes. Consistent with previous studies [28, 39, 40], current results also point out an increment in diastolic [Ca^2+^]_i_ in the MetS group. SOCE blockage (BTP2 administration) did not show any effect on diastolic [Ca^2+^]_i_. We found a significant reduction in the calcium transient amplitude of the cardiomyocytes in MetS and the amplitude was further decreased with BTP2 administration. Current results may be related to an impairment in SR filling and excitation–contraction coupling overactivation may be the underlying cause [41].

Additionally, SR Calcium-load was decreased in MetS animals but it did not change significantly after the BTP2 application. Correl et al. reported that STIM 1 overexpression increased the transient amplitude values in cardiomyocytes, while the SERCA calcium-load did not change [20], and it could affect the SERCA activity by reducing the calcium-reuptake time [30]. Therefore, MetS-induced SOCE augmentation could affect SR calcium-release and alter the RYR or SERCA activity. Besides STIM1 may detach PLN from SERCA, thereby increasing SR calcium loading in cardiomyocytes [42].

Spontaneous calcium release events (Calcium sparks) are essential for healthy and functioning cardiomyocytes [43]. We examined the effect of MetS on calcium sparks and evaluated the contribution of SOCE to spark characteristics. The amplitude of calcium sparks decreased while the frequency increased in MetS. These results are consistent with the literature [28, 39]. Since an increase in calcium leakage usually increases basal [Ca^2+^], it explains why the basal [Ca^2+^] was high in the MetS hearts. We also examined the effect of SOCE on calcium sparks via BTP2 application and revealed that SOCE blockage had decreased the amplitude and frequency of the sparks in the MetS group. Similarly, Corella et al. reported that spark amplitude decreased and the frequency increased in STIM1 overexpressing cardiomyocytes [20]. Although BTP2 application affected calcium leakage, paradoxically it did not cause any significant change in basal [Ca^2+^]. Naturally, the Na^+^/Ca^2+^ exchanger and the plasma membrane calcium ATPases are also important players in determining the basal [Ca^2+^]_i_. We could not analyze these currents this was one of the limitations of our study. Thus, further studies are needed to explain the relationship between SOCE and basal [Ca^2+^]_i_.

Fluorescence experiments using Thapsigargin showed that the increase in fluorescence intensity was much greater in MetS, and it was blunted after BTP2 application [44]. beeAccording to our data, the SOCE mechanism was evident in the MetS group, and the SOCE-related calcium influx makes an important contribution to contractility. Moreover, I_SOCE_ was insignificant in the control group which was consistent with previous findings [30]. On the contrary, Wen et al. demonstrated I_SOCE_ and F/Fo in the cells that were isolated from left ventricle tissue of 6-month-old mice [31]. The discrepancy may be due to the use of different species. Further studies are needed to conclude on this matter.

Studies showing the role of SOCE in the heart have mostly been conducted in hypertrophic hearts besides SOCE-inhibition prevented hyperthrophy development [18, 23, 45]. Multiple studies indicated the crucial role of SOCE in the development of cardiac hypertrophy. Furthermore, Parks et al. reported a significant reduction in cardiac mass in tamoxifen-restricted STIM1 knockout rats. Since STIM1 silencing alters the cardiac mass, STIM1 or SOCE mechanism could be considered a link between cardiac remodeling and calcium homeostasis [46]. In addition, Benard et al. showed that silencing STIM1 prevents hypertrophy yet still causes heart failure, therefore STIM1 silencing blocks not only malignant hypertrophy but also adaptive or physiological hypertrophy [23, 24]. In the current study, the HW/BW ratio, which is an indicator of cardiac hypertrophy did not differ among MetS and Con groups. Although we did not observe prominent hypertrophy, we observed a significant amplification in SOCE current and SOCE-related proteins which made us think that SOCE amplification precedes MetS-induced cardiac hypertrophy. On the other hand, since heart weight/body weight measurement is not the gold standart in hyperthropy evaluation, we can still speculate that there might be compensatory heart hypertrophy in MetS animals in current study. Moreover, we demonstrated cardiac hypertrophy bu using echocardiography in MetS animals in our previous study [47]. Therefore, the higher blood pressure leves despite reduced contractile force can be related to compensatory hypertrophy in cardiomyocytes.

STIM1, which is the primary component of SOCE, affects LTCC currents independent of SOCE activation [48, 49]. In the current study, LTCC currents were evaluated to reveal the effect of Mets and BTP2 application.LTCC currents did not differ between MetS and control groups; furthermore, BTP2 application did not affect the LTCC currents (Additional file 1: Figure S1).

## Conclusion

Although the role of SOCE in hypertrophic and neonatal hearts has been widely demonstrated, we could not encounter any study that concluded whether SOCE chronically regulates the levels of intracellular calcium in MetS. In our study, it has been shown that the SOCE mechanism is also activated and may contribute to the changes in the calcium mechanism.

## Supplementary Information

**Additional file 1.** Investigation of the effect of BTP2 on LTCC.

**Additional file 2.** Western blot images.

## Data Availability

The datasets generated and analysed during the current study are available in the Google Drive repository, in Graphpad prism software format (.pzf) https://drive.google.com/drive/folders/1N12n-gWZ_XLulNtuXGP_2xV64zKN6tHk?usp=sharing.
