# Supplementary figures and images for: STIM1-Orai1 interaction mediated calcium influx activation contributes to cardiac contractility of insulin-resistant rats
Source: BMC Cardiovasc Disord. 2022 Apr 5;22:147. doi: 10.1186/s12872-022-02586-w (PMC8981683; doi:10.1186/s12872-022-02586-w)

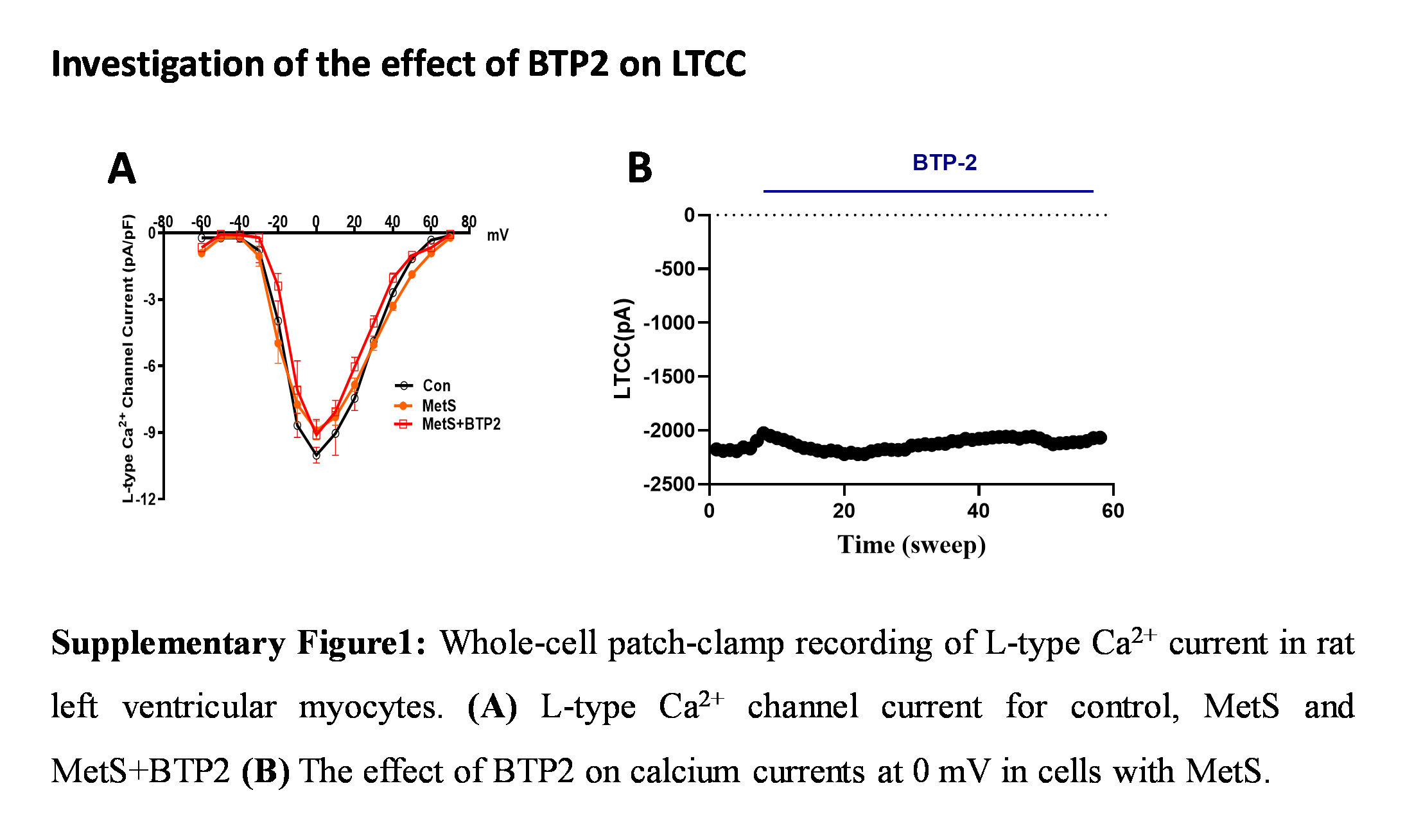

Supplement: Supplementary file 1 — Additional file 1. Investigation of the effect of BTP2 on LTCC. [file 12872_2022_2586_MOESM1_ESM.tif]

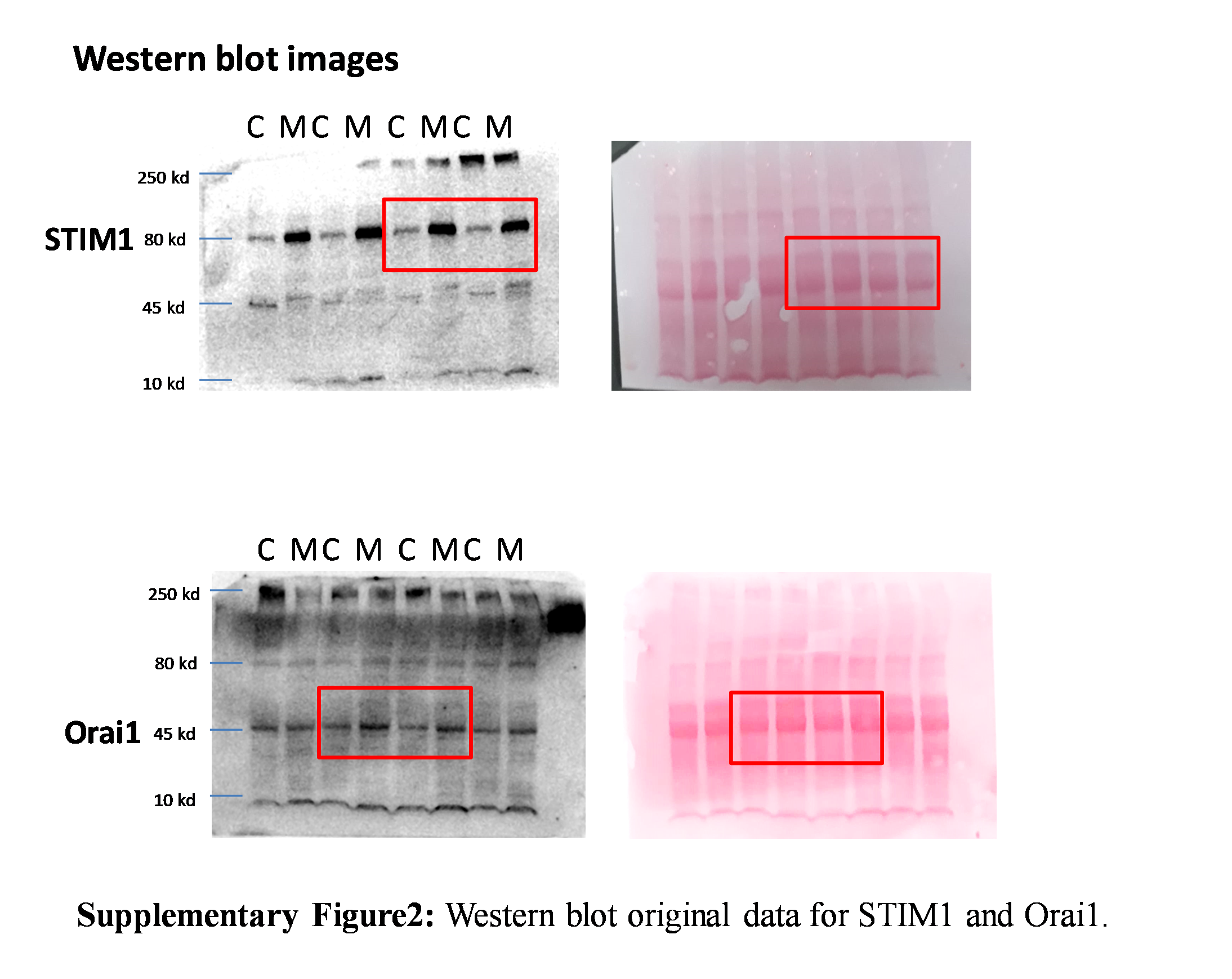

Supplement: Supplementary file 2 — Additional file 2. Western blot images. [file 12872_2022_2586_MOESM2_ESM.tif]
